# Micellar Carriers Based on Amphiphilic PEG/PCL Graft Copolymers for Delivery of Active Substances

**DOI:** 10.3390/polym12122876

**Published:** 2020-11-30

**Authors:** Justyna Odrobińska, Dorota Neugebauer

**Affiliations:** Department of Physical Chemistry and Technology of Polymers, Faculty of Chemistry, Silesian University of Technology, 44-100 Gliwice, Poland; justyna.odrobinska@polsl.pl

**Keywords:** poly(ε-caprolactone), heterografted copolymers, micellar carriers, azide-alkyne cycloaddition, Franz diffusion cells, cosmetology

## Abstract

Amphiphilic copolymers of alkyne functionalized 2-hydroxyethyl methacrylate (AlHEMA) and poly(ethylene glycol) methyl ether methacrylate (MPEGMA) with graft or V-shaped graft topologies were synthesized. The functionalization of poly(ε-caprolactone) (PCL) with azide group enabled attachment to P(AlHEMA-*co*-MPEGMA) copolymers via a “click” alkyne-azide reaction. The introduction of PCL as a second side chain type in addition to PEG resulted in heterografted copolymers with modified properties such as biodegradability. “Click” reactions were carried out with efficiencies between 17–70% or 32–50% (for lower molecular weight PCL, 4000 g/mol, or higher molecular weight PCL, 9000 g/mol, respectively) depending on the PEG grafting density. The graft copolymers were self-assembled into micellar superstructures with the ability to encapsulate active substances, such as vitamin C (VitC), arbutin (ARB) or 4-*n*-butylresorcinol (4nBRE). Drug loading contents (DLC) were obtained in the range of 5–55% (VitC), 39–91% (ARB) and 42–98% (4nBRE). In vitro studies carried out in a phosphate buffer saline (PBS) solution (at pH 7.4 or 5.5) gave the maximum release levels of active substances after 10–240 min depending on the polymer system. Permeation tests in Franz chambers indicated that the bioactive substances after release by micellar systems penetrated through the artificial skin membrane in small amounts, and a majority of the bioactive substances remained inside the membrane, which is satisfactory for most cosmetic applications.

## 1. Introduction

The selection of appropriate non-toxic, biocompatible, and biodegradable polymers that can form carriers of drugs or active substances is the most important consideration for all biomedical and cosmetological applications. The polymeric carriers are very often based on biopolymers, such as polysaccharides (i.e., cellulose [1], chitosan [2,3]), or polyesters (i.e., poly(lactic acid)) [4] and can be used safely in medical and cosmetic products. However, their synthetic substitutes have been obtained by controlled polymerizations that include grafting strategies to provide graft copolymers with nonlinear topologies and various chemical nature of the building blocks to obtain required properties [5,6,7], which can be developed by preparation of heterografted copolymers [8].

Heterografted copolymers usually contain two or more different side chain types that confer specific properties because they can be adjusted by a combination of the properly selected polymer backbone and side chains as well as their lengths and grafting degree [9]. Among the distinguishable heterografted copolymers, comb [9,10], bottle-brush [11,12,13], and tooth-brush [14] all found a wide range of applications. Heterografted brushes, which combine hydrophobic and hydrophilic side chains, show self-assembling properties in aqueous solutions that form more stable micelles as compared to analogous linear amphiphilic block copolymers and are important in designing drug delivery systems [15,16,17]. Biocompatible poly(ethylene glycol) (PEG) and biodegradable poly(ε-caprolactone) (PCL) are frequently used segments in graft copolymers, usually in combination with a poly(2-hydroxyethyl methacrylate) (PHEMA) backbone, e.g., core-shell brush with block structure of side chains PHEMA-*graft*-(PCL-*block*-PEG) [18] as well as the tooth-brush-like copolymer PEG-*block*-P(HEMA-*graft*-PCL) [19] that has been used as micellar carriers of doxorubicin. Heterografted copolymers with PEG chains are used as an (electro)active surface in biomedical applications [20], super-soft elastomeric materials [21] or stabilizers for water-in-oil emulsions [22].

The biodegradable properties of PCL are highly desired in drug delivery systems, but its hydrophobicity requires a combination with another polymer to form micellar carriers with a proper hydrophobic/hydrophilic balance. For example, delivery of anti-cancer (paclitaxel, curcumin, sulforaphane, doxorubicin) and anti-inflammatory drugs by amphiphilic copolymers based mostly on PCL/poly(ethylene glycol) (PEG) with linear topology [23,24,25], PCL/poly(acrylic acid) [26,27] or PCL/dextran [28], as well as fibers [29,30] and microspheres formed by poly(vinyl alcohol)/PCL [31] or polydimethylsiloxane/PCL [32], have been reported. PCL has also found application in gene delivery [33,34] and tissue engineering (scaffolds) [35]. In addition, PCL has been employed in delivery systems for vitamins B12 [36], E [37], A (retinol) [38], and K1 [39], and anti-viral, anti-inflammatory, anti-bacterial and antioxidant substances [40]. Because of its completely bioresorbable properties, PCL has applications in cosmetology that include skin fillers which eliminate imperfections or nanofiber wound dressings for skin regeneration [41]. An innovative and spectacular idea for hand rejuvenation with immediate, visible, and lasting effects based on PCL materials was described [42] as well as positive effect for hair growth using hinokitol-loaded PCL nanocapsules [43]. 

Here we focused on the synthesis of amphiphilic PEG graft copolymers containing PCL as the second side chain type and can be used as micellar carriers. Previously, we have described the synthesis of copolymers of alkyne functionalized 2-hydroxyethyl methacrylate (AlHEMA) and methyl methacrylate (MMA) (P(AlHEMA-*co*-MMA) and their subsequent “click” reactions with PEG using *grafting onto* strategies [44,45]. In the current work, the AlHEMA and methoxy-functionalized poly(ethylene glycol) methacrylate (MPEGMA) copolymers were obtained using atom transfer radical polymerization (ATRP). Due to the biodegradability of PCL chains, they were chosen for modification of the PEG graft copolymers to produce the (PEG/PCL) heterografted system. Their attachment was achieved by *grafting onto* technique using a “click” chemistry reaction to obtain P((HEMA-*graft*-PCL)-*co*-MPEGMA) with various degrees of PCL grafting. The self-assembly ability of these copolymers afforded an opportunity for encapsulation of selected active substances (vitamin C, VitC; arbutin, ARB; 4-*n*-butylresorcinol, 4nBRE). Those micellar systems were tested for the release of the active substance under conditions that approximate human skin (phosphate buffer saline, PBS; pH = 7.4 and pH = 5.5; 37 °C). The potential of these systems as bioactive substance carriers for cosmetology application was evaluated by permeation tests of the released substances through artificial skin using Franz chambers.

## 2. Materials and Methods

### 2.1. Materials

Poly(ethylene glycol) methyl ether methacrylate (MPEGMA, M_n_ = 500 g/mol, 97%) received from Aldrich (Poznań, Poland) while ε-caprolactone (CL, 99%), tin(II) bis(2-ethylhexanoate) (Sn(Oct)_2_, 96%), methanol (MeOH, 99%) and anisole (99%) were received from Alfa Aesar (Warsaw, Poland) and after drying were stored under nitrogen in a low temperature. Copper (I) bromide (CuBr, 98%, Fluka, Steinheim, Germany) was prepared as we reported earlier [44]. Triethylamine (TEA, 99%), 4,4-Dinonyl-2,2-dipyridyl (dNdpy, 97%), *N,N,N′,N″,N″*-pentamethyldiethylenetriamine (PMDETA, 98%), pyridine (99%), 2-bromoisobutyryl bromide (BriBuBr, 98%), ethyl α-bromoisobutyrate (EiBBr, 98%), 0.1 M sodium phosphate buffer saline (PBS; pH = 7.4), Strat-M Membrane (Transdermal Diffusion Test Model, 25 mm), and chlorotrimethylsilane (CTMS, 98%) were received from Aldrich (Poznań, Poland). Sodium azide (NaN_3_, 99%) and arbutin (ARB, 95%) were received from Acros (Geel, Belgium). Tetrahydrofuran (THF), *N,N*-dimethylformamide (DMF, 99%), anhydrous toluene (99%) and L(+)-ascorbic acid (VitC, 99%) were received from Chempur (Piekary Śląskie, Poland), 4-*n*-butylbenzene-1,3-diol (4nBRE, Ark Pharm, 95%, Gdańsk, Poland) and triethylene glycol monomethyl ether (MTEG, Fluka, 98%, Steinheim, Germany) were used as received. 2-(Prop-1-en-2-carbonyloxy)ethyl hex-5-ynate (AlHEMA), 3,7-dimethyl-9-(2,6,6-trimethylcyclohex-1-en-1-yl)nona-2,4,6,8-tetraen-1-yl 2-bromo-2-methylpropanoate (RETBr) and 4-butyl-1,3-phenylene bis(2-bromo-2-methylpropanoate) (4nBREBr_2_) were prepared according to previously reported procedures [44,45,46].

### 2.2. Characterization

NMR spectrometer a UNITY/INOVA (Varian, Mulgrave, Victoria, Australia) was operated at 300 MHz (^1^H) and 75 MHz (^13^C). The polymer samples were dissolved in dimethyl sulfoxide (DMSO) or chloroform (CDCl_3_) with tetramethylsilane (TMS). Gas chromatograph (GC, Agilent Technologies 6850 Network GC System, Santa Clara, CA, USA) was equipped with a flame ionization detector. The reaction mixture samples diluted in acetone were measured at following conditions: the injector and detector temperatures 250 °C, initial and final column temperatures of 40 °C and 200 °C, respectively. Gel permeation chromatograph (GPC, 1100 Agilent, Santa Clara, CA, USA) was equipped with an isocratic pump, degasser, thermostatic box for columns, and a differential refractometer. Polymer samples dissolved in THF were measured at 30 °C and flow rate 0.8 mL/min. Linear polystyrenes (580–300,000 g/mol) were used as a calibration standards. Spectrofluorimeter (FluoroSENS Pro-11, CAMLIN, Lisburn, Ireland) was used to obtain excitation spectra of pyrene (λ = 390 nm) as the fluorescence probe at a constant concentration (3.0 × 10^−4^ mol/L) and polymer concentrations from 5 × 10^−4^ to 1.0 mg/mL. The cross-over point in dependence of the intensity ratio (I_336_/I_332_) from the pyrene excitation spectrum vs. logC (where C is the concentration in mg/mL) was estimated a critical micelle concentration (CMC). Dynamic light scattering (DLS, Zetasizer Nano-S90, Malvern Technologies, Malvern, PA, USA) was equipped with a He-Ne laser at a fixed scattering angle (173°). Polymer aqueous solutions were measured at 25 °C in three independent runs to obtain an average value. Ultraviolet-visible light spectroscopy (UV–Vis, Thermo Fisher Scientific Evolution 300, Waltham, MA, USA) with resolution >2.0 at 0.5 nm SBW was used to analyze initial sample and the next ones taken during the release process. Scanning electron microscope (SEM, Phenom ProX, Phenom-World Bv, Eindhoven, The Netherlands) was applied to observe the micelle surface morphology for samples, which were sputtered with a layer of 5 nm gold nanoparticles. Franz diffusion cells (Teledyne Hanson Research, Phoenix DB-6, Variel Ave, Chatsworth, CA, USA) were used to evaluate in vitro permeation of active substance.

### 2.3. Synthesis of P(AlHEMA-co-MPEGMA) with 4nBREBr_2_ as Initiator (Example for V)

Initiator—4nBREBr_2_ (22.10 mg, 0.051 mmol), ligand—dNdpy (41.05 mg, 0.101 mmol), and monomers—MPEGMA (6.20 mL, 13.39 mmol) and AlHEMA (1.00 g, 4.47 mmol), as well as solvents (10 vol.% of monomers; MeOH: ANS = 1: 3): MeOH (0.180 mL), ANS (0.540 mL), were placed in a Schlenk flask. The mixture was degassed by three freeze–pump–thaw cycles and CuBr (6.40 mg, 0.045 mmol) was added. The reaction was conducted at 60 °C (using an oil bath). Air exposure stopped the polymerization. CuBr was removed using a neutral alumina column and passing through it the reaction mixture dissolved in chloroform. The polymer was precipitated by dropwise addition of a concentrated solution into diethyl ether, and then dried to constant mass. The synthetic procedures of P(AlHEMA-*co*-MPEGMA) copolymers using EiBBr or RETBr as initiators are described in Supplementary Materials (Synthesis procedure S1,S2).

### 2.4. Modification of PCL-OH to the Azide Derivative (PCL-N_3_)

PCL-OH (1.76 g, 0.22 mmol; Synthesis procedure S3) was dissolved in 70 mL of anhydrous THF to yield a colorless solution. Pyridine (18.75 µL, 0.23 mmol) was added dropwise into that mixture and cooled to 0 °C in an ice/water bath. BriBuBr (28.77 µL, 0.23 mmol) was added and stirred for 24 h at room temperature in the absence of light. The mixture was transferred into a separatory funnel with chloroform and extracted with two 100 mL portions of H_2_O. The organic phase was concentrated and the product precipitated in MeOH. The PCL-Br obtained was dried under vacuum to constant mass. Yield: 96%. ^1^H NMR (600 MHz, CDCl_3_, ppm): 4.23 (2H, -CH_2_-O(=O)C-), 4.06 (n*2H, -CH_2_-O(=O)C-), 3.66 (10H, 5* -O-CH_2_-), 3.55 (2H, -CH_2_OH), 3.38 (3H, -OCH_3_), 2.29 (2H, -CH_2_-COO-), 1.96 (2*3H, -C(CH_3_)_2_Br), 1.65 (n*4H, -CH_2_-), 1.38 (n*2H, -CH_2_-).

PCL-Br (2.87 g, 0.35 mmol) and NaN_3_ (22.48 mg, 0.35 mmol) were solved in 70 mL of anhydrous THF. The reaction proceeded for 24 h at room temperature in the absence of light. Product isolation occurred according to the above-described procedure. The product (PCL-N_3_) was dried under vacuum to constant mass. Yield: 93%. ^1^H NMR (600 MHz, CDCl_3_, ppm): 4.23 (2H, -CH_2_-O(=O)C-), 4.06 (n*2H, -CH_2_-O(=O)C-), 3.66 (10H, 5* -O-CH_2_-), 3.55 (2H, -CH_2_OH), 3.38 (3H, -OCH_3_), 2.29 (2H, -CH_2_-COO-), 1.93 (2*3H, -C(CH_3_)_2_N_3_), 1.65 (n*4H, -CH_2_-), 1.38 (n*2H, -CH_2_-). ^13^C NMR ( MHz, CDCl_3_, ppm): 172 (C7, C16, -COO-, -O(O=)C-C(CH_3_)_2_N_3_), 71-68 (C2-C5, C17, -OCH_2_-, -C(CH_3_)_2_N_3_), 63 (C12, -CH_2_-O(O=)C-), 62 (C15, -CH_2_OH), 61 (C6, -CH_2_-OC(=O)-), 58 (C1, -OCH_3_), 33 (C8, -CH_2_-C(=O)O-), 32 (C18, -C(CH_3_)_2_N_3_), 31 (C14, -CH_2_-), 27 (C11, -CH_2_-), 25 (C10, -CH_2_-), 24 (C13, -CH_2_-), 23 (C9, -CH_2_-).

### 2.5. Cu^I^-Catalyzed Azide/alkyne Cycloaddition (CuAAC) (Example for Ic_PCL_4000_)

Polymer I (1.50 g, 1.46 × 10^−2^ mmol, containing 0.849 mmol of AlHEMA units) was dissolved in THF (20 mL), and then PCL-N_3_ (PCL_4000_) (4.24 g, 0.849 mmol) and PMDETA (0.44 mL, 2.12 mmol) were added. The mixture was purified by 20 min under inert gas, and CuBr (304.40 mg, 2.12 mmol) was introduced. The reaction stirred at room temperature for 48 h in the absence of light. After removing CuBr with cationite (Dowex) the solution was concentrated. The product precipitated in MeOH was dried under vacuum.

### 2.6. Encapsulation

The amphiphilic copolymer (100 mg) and active substance (the weight ratio of copolymer: active substance = 1:1) were dissolved in methanol (15 mL), and H_2_O was added dropwise (200 vol.% of the solvent, constant mixing). The mixture was stirred for 24 h. Then MeOH was evaporated and the unloaded active substance was separated by centrifugation (6000 rpm for 10 min, 24 °C). The aqueous fraction was lyophilized by freezing to obtain a solid product. A solution of loaded micelles in PBS (0.008 mg/mL) was prepared to determine the amount of entrapped substances using UV-Vis analysis at λ = 282 for ARB, λ = 267 nm for VitC, and λ = 280 nm for 4nBRE. The absorbance of active substances was determined experimentally. Each sample was measured three times and the results were averaged. Drug loading content (DLC) was calculated as a percentage ratio of the drug loading mass to the total mass of the polymer and loaded drug.

### 2.7. Release Studies in Cellulose Membrane Bag

The solution of loaded micelles in PBS (1.0 mg/mL, pH = 7.4 or 5.5 (the pH of 0.01 M PBS was subsequently adjusted with 0.1 N HCl to pH 5.5)) was placed into a dialysis cellulose membrane bag (MWCO = 3.5 kDa) and put into a vial with PBS (50 mL, pH = 7.4 or 5.5). The release was performed in a water bath (37 °C) to ensure constant mixing. The absorbances of the released medium samples were measured by UV–Vis spectroscopy to calculate the released drug concentration. The samples were taken every 10 min for 1 h, then every 30 min until the release was complete.

### 2.8. Permeation Tests in Franz Diffusion Cells

PBS solution (15 mL) was introduced into a diffusion cell (acceptor chamber) equipped with a magnetic stirrer. The membrane and donor chamber were then placed and 1.8 mL of the test carrier solution (1.0 mg/mL) was introduced into the donor chamber. The experiment was conducted at 37 °C with continuous stirring (V = 400TPM) for 24 h. During the analysis, 200 µL of the solution was taken from the acceptor chamber at specified intervals, and the diffusion cell was supplemented with the same amount of PBS. The collected samples were analyzed by UV-Vis. Flow through the membrane (J) was calculated using the following equations:

(1) $$HLB=20\times \frac{M_{hphil.}}{M_{n}}$$

(2) $$D=\frac{e^{2}}{6\times t}$$

(3) $$J=\frac{D\times HLB\times \Delta c}{e}$$

where *HLB*—hydrophilic/lipophilic balance, *M_hphil._*—molecular weight of the hydrophilic fraction, *M_n_*—molecular weight of the copolymer, *D*—diffusion coefficient, *e*—membrane thickness, *t*—lag time, *J*—flow through the membrane, *Δc*—concentration difference on both sides of the membrane

## 3. Results

**Figure 1:**
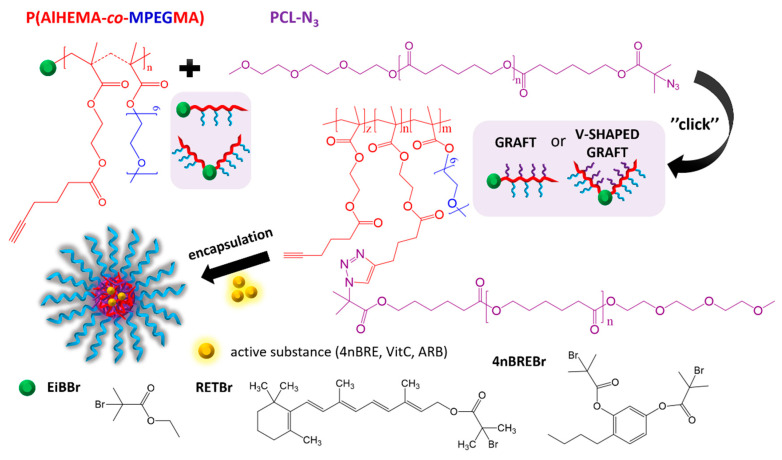
Synthesis of PEG graft copolymers with PCL as additional side chains via “click” chemistry reaction.

Controlled atom transfer radical polymerization (ATRP) was used to obtain three groups of amphiphilic PEG graft copolymers (P(AlHEMA-*co*-MPEGMA)). The polymerization *grafting through* macromonomer with the use of various ATRP initiators (standard ethyl 2-bromoisobutyryl (EiBBr), bromoester monofunctionalized retinol (RETBr), bromoester bifunctionalized 4-*n*-butylresorcinol (4nBREBr_2_)) afforded formation of PEG graft or V-shaped PEG graft copolymers (Figure 1). Varying the initial monomer proportions (AlHEMA/MPEGMA: 25/75, 50/50) to control the copolymer hydrophilicity and the alkyne group content was crucial for the subsequent “click” reaction. 

**Figure 2:**
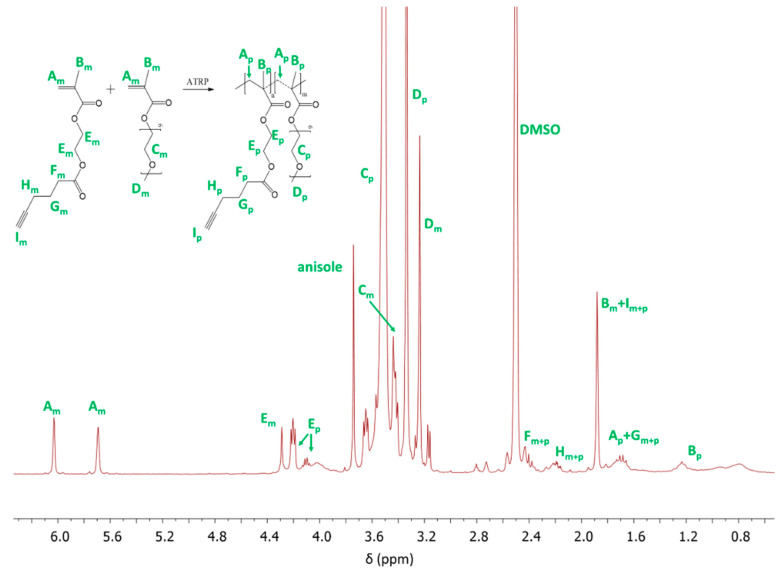
^1^H NMR spectrum of the reaction mixture for copolymerization of AlHEMA/MPEGMA (I), where m and p indicate the resonances for monomer and polymer, respectively.

^1^H NMR analysis allowed to confirm the structure of copolymers (I-VI) (Figure 2). The resonances at 0.6–1.5 ppm (B_p_) represent -CH_3_ group protons in the main copolymer chain, and the other signals are assigned to those from the -OCH_2_CH_2_O- groups in side chains of MPEGMA units (C_p_: 3.51 ppm) and for the -COOCH_2_CH_2_OCO- groups in AlHEMA units (E_p_: 3.86–4.24 ppm). 

Monomer conversion was determined by GC analysis to calculate the polymerization degree (DP_n_), molecular weight (M_n_), and grafting degree (DG_PEG_). Generally, polymerizations were completed with (macro)monomer conversions from 35–60% for AlHEMA and 24–60% for MPEGMA yielding DP_n_ levels mostly above 170 and DG_PEG_ values that ranged from 34–80% (Table 1). Comparable conversions of AlHEMA vs. MPEGMA (I-IV) suggested the formation of copolymers with a statistical distribution of PEG grafts, whereas, in graft V-shaped copolymers (V-VI), the lower macromonomer conversion was responsible for the gradient structure with an abundance of MPEGMA units. These polymer structure differences were caused by the impact of the initiator type, e.g., monofunctional vs. bifunctional. The bifunctional initiator generated two active centers that appeared to favor the low molecular weight monomer during chain propagation. A comparison of each series with varying the initial AlHEMA/MPEGMA proportions indicated that the equimolar comonomer combination (50/50) resulted in lower MPEGMA conversions (II and IV) than for mixtures with a dominant macromonomer (I and III), yielded shorter polymeric chains and a lower grafting degree (DG_PEG_ = 56% vs. 80%). Polymerization systems with a bifunctional initiator were exceptions to this behavior and showed the opposite behavior that was likely due to the lower reactivity of MPEGMA, which significantly reduced the reaction rate when present in higher amounts and gave the shortest chains for polymer V (DP_n_ = 114) for similar reaction times relative to VI. In the latter case, the obtained polymer VI was characterized with a relatively long backbone but the lowest PEG grafting degree (DG_PEG_ = 34%). 

**Figure 3:**
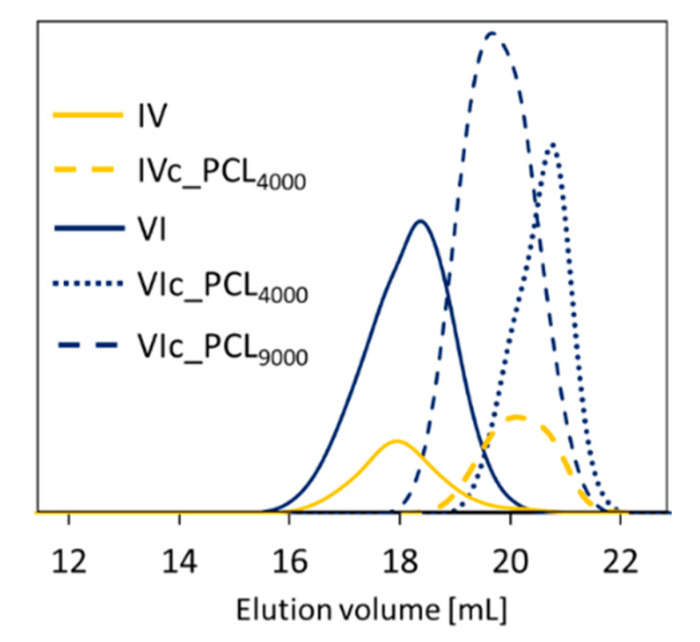
GPC traces of representative PEG graft and PEG/PCL heterograft copolymers.

The comparatively low dispersity indices (Đ) illustrated by symmetrical and monomodal signals of GPC traces (1.28–1.44, Table 1, Figure 3 solid line) confirmed narrow formation of homogeneous polymer chains as the effects of the well-controlled ATRP. The predominance of AlHEMA units in copolymers II, IV, VI (55–66%) corresponded to a slight broadening of the GPC signal and higher dispersity (1.76–2.18) and suggested an increased sensitivity of the polymerization systems to the occurrence of side reactions. The hydrodynamic volume of graft copolymer in solution was lower than the linear polymer standards used in GPC calibration, which explains the discrepancy between the M_n_ values calculated from conversion by GC and those determined by GPC analysis that provided the apparent molecular weight values.

The parallel modification of the hydroxyl group to the azide group in PCL benefited the copper-catalyzed azide/alkyne cycloaddition (CuAAC) as the “click” reaction for introduction of the second type of side chain and formation of heterograft copolymers (Figure 1). For this purpose, PCLs with two different molecular weights (~4000 and ~9000 g/mol) and low dispersity indices (1.25–1.29) were synthesized by ring-opening polymerization (ROP; Table 2). The hydroxy terminated PCLs were subjected to an esterification reaction to introduce a bromoester group, which was then converted into an azide via substitution of the bromine atom. Formation of the bromoester and the azide derivatives was observed by ^1^H NMR, which detected the appearance of signals from the methyl protons on the isobutyrate group (H_J_: 1.96 ppm, Figure S1b) and its downfield shifts (H_J_: 1.93 ppm, Figure S1c) after azidation. The final products were also characterized by ^13^C NMR, where a methyl group signal (Figure S2b: C18 at 32 ppm), as well as the signal from the carbon directly connected to the azide group (Figure S2b: C17 at 70 ppm) were detected. The modified polymers were characterized by low dispersity and narrow GPC traces (Table 2, Figure S3). 

**Figure 4:**
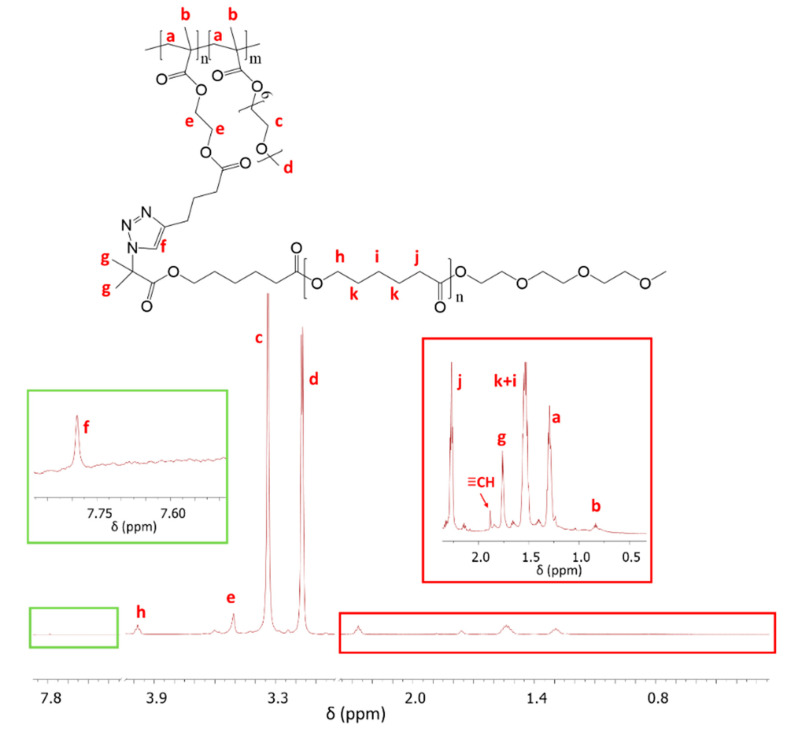
^1^H NMR spectrum of PEG heterograft copolymer with short PCL side chains (Ic_PCL_4000_).

The azide derivatives of PCL_4000_ or PCL_9000_ were *grafted onto* P(AlHEMA-*co*-MPEGMA)s by alkyne groups as the conjugation sites via a Huisgen “click” chemistry CuAAC reaction, which led to heterografted copolymers P((HEMA–*graft*–PCL)–*co*–MPEGMA) (Figure 1) with amphiphilic and biodegradable properties. Variation of PCL side chain length (PCL_4000_ vs. PCL_9000_) was an additional parameter to adjust the ability of the heterograft copolymers to self-assemble into micellar structures for encapsulation of active substances. The presence of a triazole proton at 7.80 ppm (H_f_) in the ^1^H NMR of heterograft copolymer Ic_PCL_4000_ (Figure 4) confirmed the “click” chemistry attachment of PCL side chains. Additional signals at 3.98 ppm (H_h_), 2.26 ppm (H_j_), 1.76 ppm (H_g_), and 1.51 ppm (H_k+i_) came from the PCL grafts, whereas the signals at 3.51 ppm (H_e_), 3.34 ppm (H_c_), 3.17 ppm (H_d_), 1.28 ppm (H_a_), 0.82 ppm (H_b_) corresponded to protons in the main polymer chain. Chemical structures of the PEG/PCL graft copolymers were also confirmed by ^13^C NMR (Figure S4), which detected the signals from the triazole ring carbon (C13 at 133 ppm), PCL side chain carbons (C7 at 49 ppm and C1, C11 at 182 ppm) and in the main chain (C8 at 70 ppm, C6 at 64 ppm, C12 at 173 ppm).

**Figure 5:**
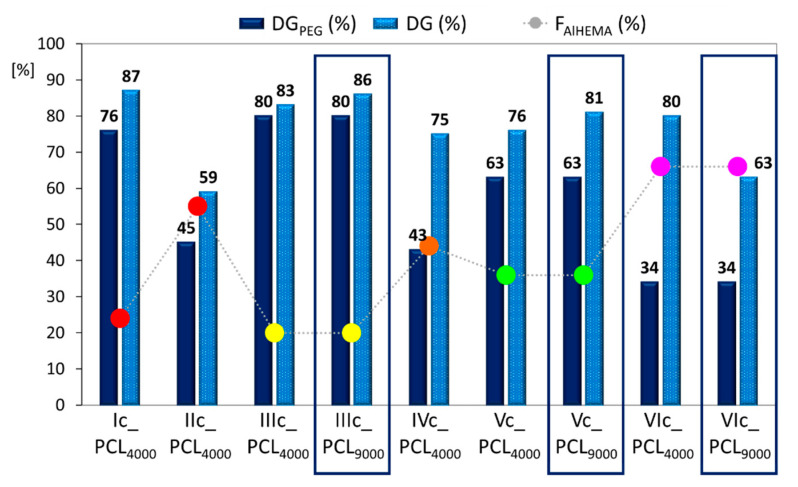
Effect of F_AlHEMA_ and PCL length on the degree of copolymer grafting increase, where DG_PEG_—degree of PEG grafting before “click” reaction; DP—total degree of grafting after PCL “click”.

The effectiveness of the “click” chemistry reaction (E_click_, Table 3) was calculated as a percentage from the integration of resonances corresponding to the hydrogen signal in the triazole ring (H_f_: 7.80 ppm) relative to the sum of integration signals assigned to unreacted alkyne groups (H≡CH: 1.88 ppm). The PCL “click” was performed over a broad range from 17–70%, including 32–50% for the high molecular weight PCL. The “click” effect of various length PCLs *grafted onto* the same polymer matrix with hydrophilic levels above 60% (F_PEGMA_) indicated the following ordering, PCL_4000_ < PCL_9000_, but it was reversed at a lower hydrophilic fraction level (E_click_ PCL_4000_/PCL_9000_ = 17/32% IIIc and 36/50% Vc vs. 70/44% VIc) (Table 3). These results influenced changes in the degree of copolymer grafting (DG_PEG_ vs. DG, Figure 5), showing moderate benefits from varying the PCL molecular weight (83/86%, 76/81%, 80/63%). The significant DG increase was supported by VIc_PCL_4000_ and VIc_PCL_9000_ with the highest F_AlHEMA_ level and V-shaped graft topology (DG/DG_PEG_ = 2.4 and 1.9, respectively) and, which is reasonable for the lowest content of PEG grafts. This means the creation of carriers with greater biodegradability due to the higher number of PCL side chains was favored by *grafting onto* the PEG graft copolymer with lower hydrophilicity, which in this case was supported by the low number of MPEGMA units that reduced the steric hindrance and promoted by the V-shaped structure. After PCL grafting, the dispersity indices values (Table 3) were lower or comparable to their precursors P(AlHEMA-*co*-MPEGMA)s, which were also presented by monomodal GPC signals (Figure 3). 

The self-assembling behaviors of the graft copolymers were investigated by determining their critical micelle concentration (CMC) using a fluorescence spectrometry. Data from the emission spectra of pyrene yielded a plot of I_336_/I_332_ vs. the log of copolymer solution concentration (Table 3, Figure S5). The plot indicated PCL molecular weight clearly influenced the micellization capacity of the copolymer. Including the hydrophilicity level of the copolymer (F_hphil._), adjusted by the degree of grafting PEG/PCL as well as the length of PCL grafts (PCL_4000_ vs. PCL_9000_), the presence of the latter decreased the CMC (IIIc: 0.0254 vs. 0.0080 mg/mL, Vc: 0.1641 vs. 0.0355 mg/mL, VIc: 0.0070 vs. 0.0027 mg/mL) due to higher hydrophobicity; that means that micellization occurred at a lower concentration than the PCL_4000_ systems. Similarly, comparing the systems in each series with the same length of PCL, the reduced PEG/PCL ratio generated higher hydrophobicity (e.g., Ic vs. IIc: 0.0136 vs. 0.0033 mg/mL). The same correlation was observed for V-shaped graft copolymers, but the CMC values were higher at the comparable level of hydrophobicity for graft copolymers (e.g., Vc vs. Ic or IIc), whereas comparable CMC values were detected at significantly higher hydrophobic fraction (>90% for both VIc systems vs. 60–80% for IIc, IVc, and IIIc_PCL_9000_). These results confirmed that micelles are more readily formed by more hydrophobic systems; however, topology, i.e., graft vs. V-shaped graft, is also an important factor.

**Figure 6:**
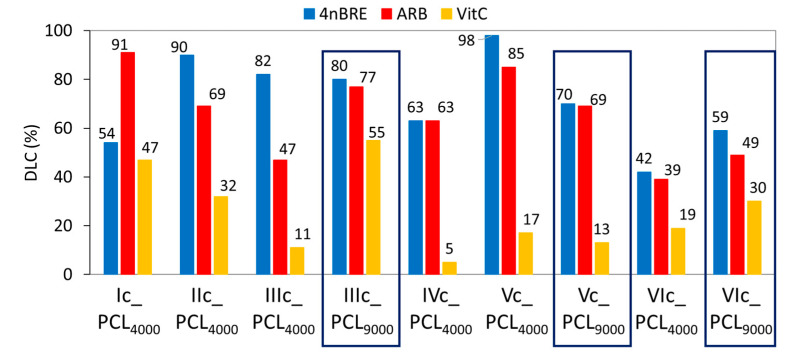
DLC for micelles formed by PEG graft copolymers with PCL_4000_ and PCL_9000_.

The self-assembly ability of the amphiphilic copolymers was assisted the formation of micellar carriers loaded with select bioactive substances. A retinol derivative, 4nBRE, is widely used in cosmetic products such as anti-wrinkle creams and to reduce discoloration. ARB benefits are mainly related to a reduction of skin inflammation and discoloration, while VitC with antioxidant properties stimulates collagen synthesis. The encapsulation efficiency (polymer:drug = 1:1) was characterized by the drug loading content (DLC, Figure 6) using UV–Vis spectroscopy. In almost all cases, VitC was encapsulated with the lowest efficiency (5–55%) as compared to ARB (39–91%) and 4nBRE (42–98%). The reduced hydrophilicity of the graft copolymer, generated by longer PCL grafts (e.g., IIIc_PCL_4000_ vs. IIIc_PCL_9000_ with F_hphil_: 68 vs. 39, respectively), resulted in a higher DLC, but for Vc, this effect was reversed because the PEG/PCL ratio was significantly lower due to a decrease in PCL chains. However, the hydrophilicity reduced by DP_PEG_ in each series was usually disadvantageous to improve DLC. The encapsulation efficiency is due to the hydrophilic/hydrophobic balance of the copolymer in which the PCL side chain lengths and the DG may adjust DLC levels to change the nature of amphiphilicity.

**Figure 7:**
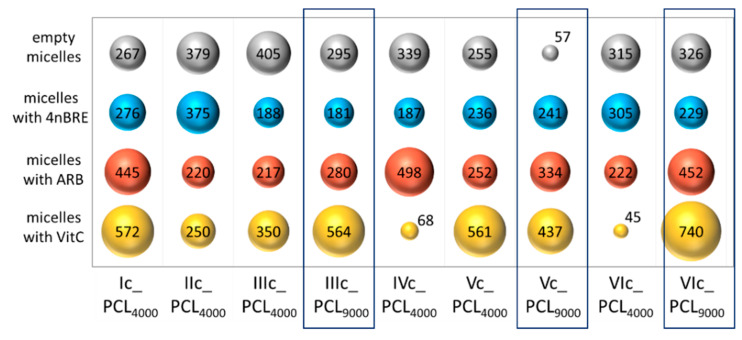
Hydrodynamic diameters (nm) by intensity for the obtained micelles.

The hydrodynamic diameters (D_h_) of the self-assembling particles were determined by DLS in an aqueous solution (Table S1). The analysis indicated formation of homogeneous particles and monomodal signals (Figure S6). Most of the micelles reached diameters between 100–300 nm (Figure 7). The influence of the PCL molecular weight on the hydrodynamic diameter of the empty micelles was detected as the size reducing effect supported by long PCL side chains. It has been noted that despite the smallest DLC values achieved by VitC micelles, (i.e., IVc_PCL_4000_, VIc_PCL_4000_) they have diameters above 400 nm in most cases. The hydrodynamic diameter increase of the encapsulated micelles followed the order: 4nBRE < ARB < VitC (Figure 7), and mimicked the encapsulation effect, which may be related to the hydrophilicity difference between the micelle-forming copolymer and the active substance, including its loaded amount.

**Figure 8:**
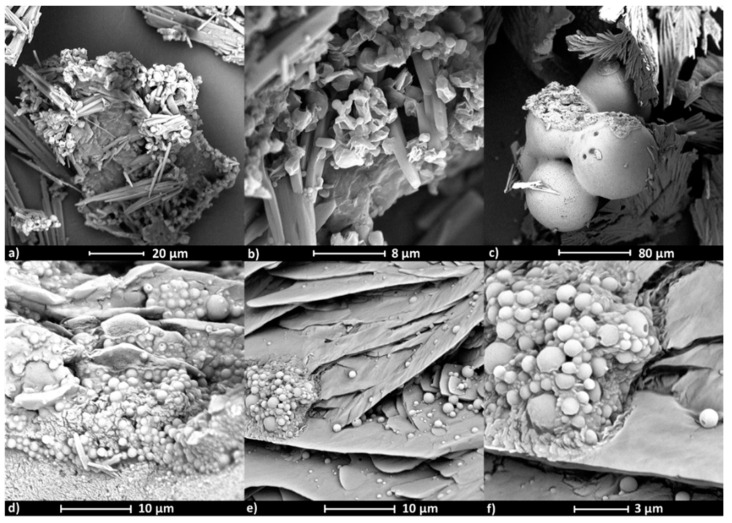
SEM images of micelles formed by the heterografted copolymer PEG/PCL_9000_ (IIIc_PCL_9000_) with encapsulated ARB (**a**,**b**) and VitC (**c**–**f**).

**Figure 9:**
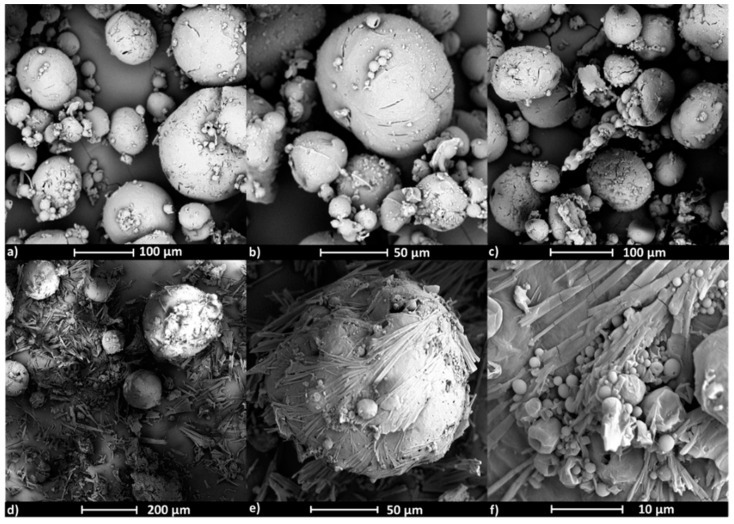
SEM images of micelles formed by the heterografted copolymer PEG/PCL_4000_ (VIc_PCL_4000_), empty (**a**–**c**) or encapsulated with ARB (**d**–**f**).

SEM images show the influence of the active substance type on the polymer micelle morphology (Figure 8 and Figure 9). Micelles formed by copolymer IIIc_PCL_9000_ (PEG/PCL_9000_) containing ARB (Figure 8a,b, Figure S7a) have rectangle and cylindrical shapes, while micelles loaded with VitC are spherical as single objects; though at larger concentrations can form aggregates (Figure 8c–f, Figure S7b,c), which correlates to the larger hydrodynamic diameters (>400 nm) detected by DLS measurements. Similar effects were observed for VIc_PCL_4000_ (PEG/PCL_4000_), where empty micelles were spherical though slightly flattened with a rather smooth surface that created clusters (Figure 9a–c, Figure S7d). After ARB encapsulation, the micelles became perfectly spherical and their smooth surface was covered with thin needle forms (Figure 9d–f, Figure S7e,f).

**Figure 10:**
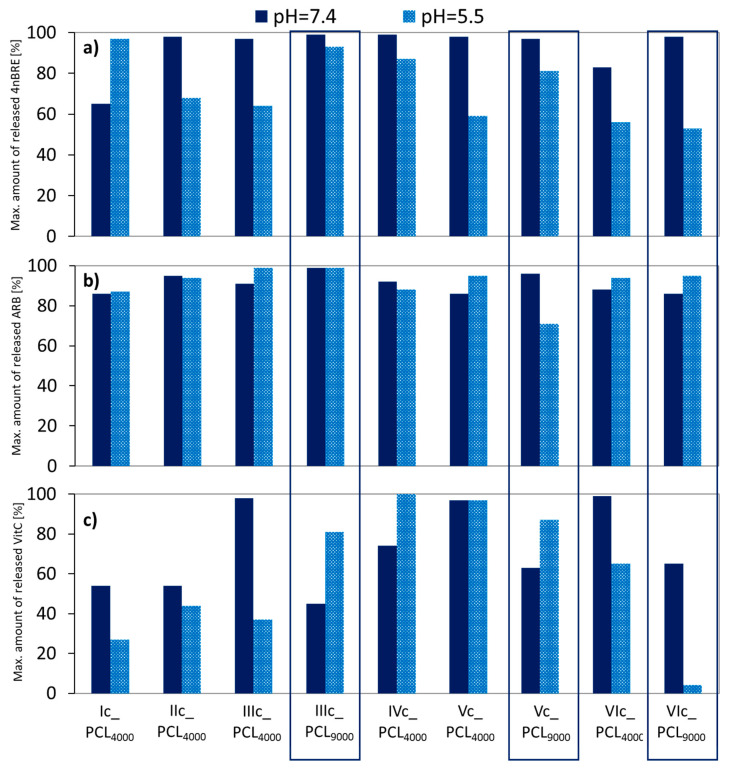
Maximum amount of released (**a**) 4nBRE, (**b**) ARB, and (**c**) VitC in pH = 7.4 and 5.5.

Release experiments were carried out in PBS at pH = 7.4 and 5.5. The lower pH more closely approximates that on the face skin, to which the described micellar systems are dedicated. Both 4nBRE and ARB released almost completely (>90%) in pH = 7.4 (Figure 10a,b). At a lower pH, the amount of released 4nBRE decreased to 60%, but there was no impact of pH on the release of ARB. At both pH levels, more VitC was released from micelles formed by the V-shaped graft copolymers (Vc_PCL_4000_/Vc_PCL_9000_/VIc_PCL_4000_ DLC = 13–19%), and graft copolymers IIIc_PCL_4000_ and IVc_PCL_4000_ (DLC = 11% and 5%), but the latter one was not taken into account due to low profitability for use in cosmetic products.

**Figure 11:**
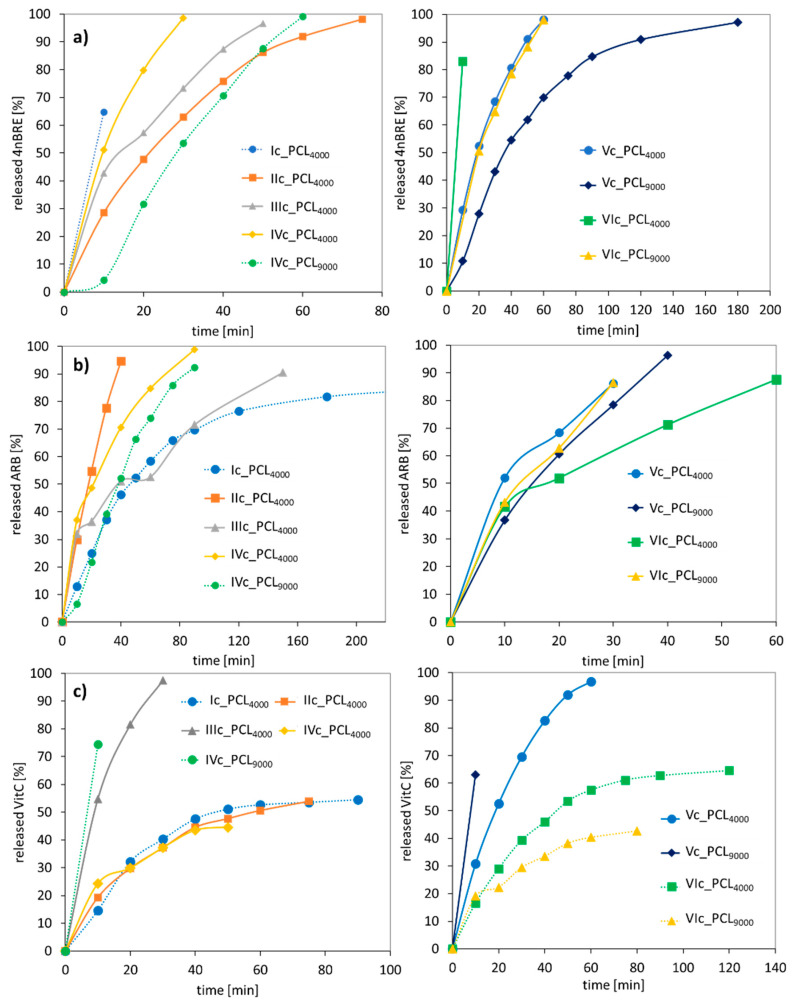
Kinetic profiles for (**a**) 4nBRE, (**b**) ARB, and (**c**) VitC released from polymer micelles at pH = 7.4.

The kinetic profiles at pH = 7.4 demonstrated the “burst” release within the first hour of release, reaching 60–100% for 4nBRE, 80–100% for ARB, and 40–100% for VitC (Figure 11). VitC is hydrophilic and slightly acidic which favored a slower release; however, 4nBRE and ARB are less hydrophilic and have a higher pH and favored an accelerated release. However, at lower pH, the effect was just the opposite, slower release rates for 4nBRE and ARB (40–100% within 1 h) and VitC needed less than an hour for maximum release (Figure S8). The differences in release profiles suggested the advantage of the “tailor-made” polymer carriers that could release the active substance under the appropriate conditions, which would depend on the specific use of the cosmetic or medical product. Comparing these micellar carriers with previously described conjugates also based on AlHEMA/MPEGMA copolymers, the latter ones bound less to the active substance, i.e., ferulic or lipoic acid (13–38%) [47]. Moreover, it took longer, up to 4 h, to release the usually lower amount of the conjugated bioactive molecules (~50%) than in the case of micelles. The unquestionable advantage of conjugates is their smaller size, which makes it possible to use them externally on the skin surface, but also as products that penetrate the skin.

Because of the above-described parameters, micelles created from copolymers IIIc_PCL_4000_, IIIc_PCL_9000_ and Vc_PCL_4000_, Vc_PCL_9000_ were selected for additional permeability tests through artificial skin using Franz cells. Studies have shown high amounts of the released active substance, mainly 4nBRE and VitC (^FC^R_max_ > 60%) during the first 60 min in most of those systems (Table 4). ARB was released at slightly lower levels (^FC^R_max_ = 36–42%), but it diffused through an artificial skin membrane into the solution more efficiently than the other substances (SOL_AS_ = 12–26% vs. 1–9%), except for micelles IIIc_PCL_4000__4nBRE, where upwards 50% of the released substance diffused into solution. However, a large part of the released substance remained in the membrane (ultimately in the skin), and only a small amount penetrated through it (Figure S9). This behavior is acceptable from a cosmetology application point of view (masks, eye pads), in which the polymer delivery system becomes immobilized, and the active substance released acts locally on the skin surface or in its superficial layers but does not penetrate deeper into the body. The problem of removing the polymer carrier from the body is solved in this case. The influence of the hydrophilic/lipophilic balance (HLB) driven by the length of the PCL side chains on the permeation rate of the active substance through the membrane (J) was observed for V-shaped graft copolymers. The lower HLB for Vc_PCL_9000_ vs. Vc_PCL_4000_ (3.19 vs. 6.81) was associated with a lower permeation rate (J[(µg/cm^2^)/h]: 47.56 vs. 22.65, 83.14 vs. 34.97, 1.63 vs. 0.27). This means a higher proportion of the hydrophilic fraction in the copolymer formed the carrier can be used to accelerate diffusion through the membrane. The highest permeation parameter values were obtained for the ARB loaded micelles in each series and showed their great potential in active substance delivery for deeper penetration into skin layers (Table 4).

## 4. Conclusions

The heterograft copolymers P(HEMA-*graft*-PCL)-*co*-MPEGMA), which varied by grafting degree, PEG/PCL side chain ratio and PCL graft length (PCL_4000_ or PCL_9000_), were designed for delivery substances applicable in cosmetology that include dermatological problems. All synthesized amphiphilic graft copolymers with varying hydrophilic-hydrophobic balances showed self-assembly ability indicated by CMC values and observed by SEM. Most of the polymers provided micellar particles with hydrodynamic diameters from 100–300 nm, but the larger particle sizes do not exclude these systems from potential application as they can be used externally on the skin surface without the need to enter the body to release the active substance. Aqueous self-assembly was used to encapsulate the selected cosmetic substances (ARB, 4nBRE, VitC) into micelles with relatively high efficiencies for almost all systems (DLC > 60%), except for VitC (5–55%). In vitro studies carried out in a PBS solution (pH 7.4 or 5.5) demonstrated the maximum release of active substances (>70%) was achieved within the first hour. However, the nature of the active substance interacted with polymer matrix varied by biodegradable PCL graft length and hydrophilic PEG graft levels was a crucial difference in the release profiles, which also depended on the solution pH. The permeation tests of the active substance released through a membrane acting as human skin in Franz chambers indicated moderate diffusion into solution and remained in the membrane (VitC, 4nBRE). The best permeation rate results were observed for ARB. Future investigations of the synthesized polymer carriers with desirable properties (high antioxidant level, satisfactory levels of the released substance in a relatively short time, and controlled release profile) will focus on the evaluation of their biochemical potential that includes cytotoxicity and possible anti-aging effects examining β-galactosidase activity. 

## Figures and Tables

**Table 1:** Data for AlHEMA/MPEGMA copolymers synthesized by ATRP.

No.	M1/M2	Time [h]	Conversion ^a^ (%)		DG_PEG_^a^ (%)	DP_n_^a^	M_n_^a^ (g/mol)	M_n_^b^ (g/mol)	Đ^b^
			M1	M2					
I	25/75	2.5	58	60	76	238	102,500	54,400	1.29
II	50/50	2.0	51	41	45	184	64,000	50,500	2.18
III	25/75	4.0	35	46	80	173	76,600	61,700	1.44
IV	50/50	3.0	35	44	56	158	59,600	57,000	1.76
V	25/75	5.0	41	24	63	114	45,800	23,500	1.34
VI	50/50	4.0	60	31	34	182	58,200	47, 600	1.88

Conditions: [M1 + M2]_0_/[In]_0_/[CuBr]_0_/[dNdpy]_0_ = 400/1/1/2.25, anisole/methanol = 9:1 10 vol.% mon; 60 °C, where: M1 is AlHEMA and M2 is MPEGMA; In is EiBBr (I-II), RETBr (III-IV), 4nBREBr_2_ (V-VI), ^a^ calculated with the use of conversion by GC analysis; ^b^ determined by GPC in THF with polystyrene standards; DG_PEG_ is grafting degree of PEG; D_n_—degree of polymerization.

**Table 2:** Data for PCL synthesis and modifications.

Polymer		DP_PCL_^c^	M_n_^c^ (g/mol)	M_n_^d^ (g/mol)	Đ^d^
PCL_4000_	^a^ PCL-OH	32	4000	5700	1.25
	PCL-Br		4100	5600	1.28
	PCL-N_3_		4050	5400	1.30
PCL_9000_	^b^ PCL-OH	76	8660	8700	1.29
	PCL-Br		8810	9300	1.27
	PCL-N_3_		8770	8900	1.31

Conditions: ^a^ [CL]_0_/[MTEG]_0_/[Sn(Oct)_2_]_0_ = 50/1/0.1, ^b^ [CL]_0_/[MTEG]_0_/[Sn(Oct)_2_]_0_ = 25/1/0.1, ^a,b^ toluene = 30 vol.% mon; 100 °C; 24 h, ^c^ calculated with the use of NMR analysis, ^d^ determined by GPC in THF with polystyrene standards.

**Table 3:** Characteristics of “click” graft copolymers P((HEMA–*graft*–PCL)–*co*–MPEGMA).

No.	F_AlHEMA_ (%)	E_click_^a^ (%)	n_tria_^a^	DG_PCL_ (%)	F_hphil._ (%)	M_n_^a^ (g/mol)	Đ^b^	CMC ^c^ (mg/mL)
Ic_PCL_4000_	24	45	26	11	43	207,500	1.39	0.0136
IIc_PCL_4000_	55	25	26	14	24	168,700	1.40	0.0033
IIIc_PCL_4000_	20	17	6	3	68	100,900	1.23	0.0254
IIIc_PCL_9000_		32	11	6	39	176,300	1.29	0.0080
IVc_PCL_4000_	44	43	30	19	24	180,900	1.40	0.0029
Vc_PCL_4000_	36	36	15	13	34	106,400	1.41	0.1641
Vc_PCL_9000_		50	20	18	16	227,000	1.50	0.0355
VIc_PCL_4000_	66	70	84	46	8	397,200	1.26	0.0070
VIc_PCL_9000_		44	53	29	6	537,900	1.46	0.0027

F_AlHEMA_—content of AlHEMA in the copolymer; E_click_—efficiency of “click” reaction; n_tria._—number of triazole ring in the copolymer; DG_PCL_—grafting degree of PCL after “click” reaction; F_hphil._—content of hydrophilic fraction; ^a^ calculated with the use of NMR analysis, ^b^ determined by GPC in THF with polystyrene standards, ^c^ determined by fluorescence spectrophotometry.

**Table 4:** Data for permeation tests in Franz chamber cells.

No.	DLC (%)	DLC_apr_ (%)	^FC^R_max_ (%)	SOL_AS_ (%)	MEM_AS_ (%)	T_max_ (min)	$D\;(\frac{cm^{2}}{h})\times 10{-}^{4}$	HLB	$J\;\left(\frac{\frac{µg}{cm^{2}}}{h}\right)$
IIIc_PCL_4000__4nBRE	82	22.15	73	50	23	15	6.0	13.56	57.86
IIIc_PCL_4000__ARB	47	29.09	38	12	26	45	7.5	13.56	98.35
IIIc_PCL_4000__VitC	11	0.47	96	6	90	25	6.0	13.56	1.25
IIIc_PCL_9000__4nBRE	80	4.14	95	4	91	90	7.5	7.76	7.96
IIIc_PCL_9000__VitC	55	3.48	94	1	93	30	7.5	7.76	6.75
Vc_PCL_4000__4nBRE	98	34.95	65	4	61	25	6.0	6.81	47.56
Vc_PCL_4000__ARB	85	48.99	42	22	20	45	7.5	6.81	83.14
Vc_PCL_4000__VitC	17	0.97	94	6	88	120	7.5	6.81	1.63
Vc_PCL_9000__4nBRE	70	35.55	49	5	44	15	6.0	3.19	22.65
Vc_PCL_9000__ARB	69	44.17	36	26	10	20	7.5	3.19	34.97
Vc_PCL_9000__VitC	13	0.43	97	9	88	180	6.0	3.19	0.27

DLC—drug loading content in carrier before permeation test, DLC_apr_—drug loading content in carrier after permeation test, ^FC^R_max_—amount of released substance using Franz cells, T_max_—time of permeation test after which the concentration of the substance in the solution did not increase, D—diffusion coefficient, HLB—hydrophilic/lipophilic balance, J—flow through the membrane, SOL_AS_—amount of release substance, which passed through the membrane into the solution, MEM_AS_—amount of release substance that remained in the membrane.

## Supplementary Materials

The following are available online at https://www.mdpi.com/2073-4360/12/12/2876/s1, Procedures for synthesis of P(AlHEMA-*co*-MPEGMA) with EiBBr and RETBr initiator (S1, S2), PCL-OH (S3). Table S1. Detailed data for DLS analysis of micelles. Figure S1. ^1^H NMR spectra of PCL-OH, PCL-Br, and PCL-N_3_. Figure S2. ^13^C NMR spectra of PCL-OH, and PCL-N_3_. Figure S3. GPC traces for PCL_4000_ before and after modifications. Figure S4. ^13^C NMR of IVc_PCL_4000_. Figure S5. Representative plots of intensity I_336_/I_332_ ratios as a function of the logarithm of copolymers concentration in aqueous solution (a) and excitation spectra of pyrene in aqueous solutions (b). Figure S6. Size distribution intensity plots for empty and loaded micelles. Figure S7. SEM images for empty and loaded micelles. Figure S8. Kinetic profiles for 4nBRE, ARB, and VitC released from polymer micelles at pH = 5.5. Figure S9. Graph of the amount of unreleased substance (NR), amount of released substance which passed through the membrane into the solution (SOL_AS_), and amount of released substance that remained in the membrane (MEM_AS_).
