# Supplementary material for: Micellar Carriers Based on Amphiphilic PEG/PCL Graft Copolymers for Delivery of Active Substances
Source: Polymers (Basel). 2020 Nov 30;12(12):2876. doi: 10.3390/polym12122876 (PMC7760728; doi:10.3390/polym12122876)
Supplement: Supplementary file 1 [file polymers-12-02876-s001.pdf]

polymers

Supporting Information

Article

Micellar Carriers Based on Amphiphilic PEG/PCL Graft Copolymers for Delivery of Active Substances

Justyna Odrobińska and Dorota Neugebauer *

Department of Physical Chemistry and Technology of Polymers, Faculty of Chemistry, Silesian University of Technology, 44-100 Gliwice, Poland; justyna.odrobinska@polsl.pl
* Correspondence: dorota.neugebauer@polsl.pl

Received: 11 November 2020; Accepted: 29 November 2020; Published:

Content:

Synthesis procedure S1. Synthesis of P(AlHEMA-co-MPEGMA) with EiBBr as Initiator (Example for I).

Synthesis procedure S2. Synthesis of P(AlHEMA-co-MPEGMA) with RETBr as Initiator (Example for III)

Synthesis procedure S3. Synthesis of PCL-OH

Table S1. Hydrodynamic diameters (D_h) for obtained micelles.

Figure S1. ^{1}H NMR spectra of (a) PCL-OH, (b) PCL-Br, and (c) PCL-N_3.

Figure S2. ^{13}C NMR spectra of (a) PCL-OH, (b) PCL-N_3.

Figure S3. GPC traces for PCL_{4000} before and after modifications.

Figure S4. ^{13}C NMR of IVc_PCL_{4000}.

Figure S5. Representative plots of intensity I_{336}/I_{332} ratio as a function of the logarithm of copolymers concentration in aqueous solution (a) and excitation spectra of pyrene in aqueous solutions (λ = 390 nm) in dependence of IIIc_PCL_{4000} copolymer concentration (b).

Figure S6. Size distribution intensity plots for micelles formed by (a) IIIc_PCL_{9000}, and (b) VIc_PCL_{4000}. Figure S7. SEM images for micelles formed by (a) IIIc_PCL_{9000} copolymer with arbutin, (b, c) IIIc_PCL_{9000} copolymer with vitamin C, (d) VIc_PCL_{4000} copolymer (empty micelles) and (e, f) VIc_PCL_{4000} copolymer with arbutin.

Figure S8. Kinetic profiles for (a) 4nBRE, (b) ARB, and (c) VitC released from polymer micelles at pH=5.5.

Figure S9. Graph of the amount of unreleased substance (NR), amount of released substance which passed through the membrane into the solution (SOL_{AS}), and amount of released substance that remained in the membrane (MEM_{AS}).

Synthesis procedure S1. Synthesis of P(AlHEMA-co-MPEGMA) with EiBBr as Initiator (Example for I)

dNdpy (41.05 mg, 0.101 mmol), MPEGMA (6.20 mL, 13.39 mmol), AlHEMA (1.00 g, 4.46 mmol), and solvents (10 vol.% of monomers; MeOH : ANS = 1: 6): MeOH (0.103 mL), ANS (0.612 mL) were placed in a Schlenk flask and degassed by two freeze–pump–thaw cycles. Then, EiBBr (6.62 µL, 0.045 mmol) was added and degassed again. After that, CuBr (6.40 mg, 0.045 mmol) was added. The reaction flask was immersed in an oil bath at 60 °C. The polymerization was stopped by exposure to air. Then, the mixture was dissolved in chloroform and passed through a neutral alumina column to remove CuBr. The solution was concentrated and the polymer was precipitated by dropwise addition of a concentrated solution into diethyl ether. The product was isolated by decantation and dried under vacuum to constant mass.

Synthesis procedure S2. Synthesis of P(AlHEMA-co-MPEGMA) with RETBr as Initiator (Example for III)

RETBr (19.42 mg, 0.045 mmol), dNdpy (41.00 mg, 0.100 mmol), MPEGMA (6.20 mL, 13.39 mmol), AlHEMA (1.0 g, 4.47 mmol), and solvents (10 vol.% of monomers; MeOH : ANS = 1: 3): MeOH (0.180 mL), ANS (0.540 mL) were placed in a Schlenk flask and then degassed by three freeze–pump–thaw cycles. After that, CuBr (6.40 mg, 0.045 mmol) was added. The reaction flask was immersed in an oil bath at 60 °C. The next steps were performed according to above-described procedure for the synthesis of P(AlHEMA-*co*-MMA) with EiBBr (Synthesis procedure 1).

Synthesis procedure S3. Synthesis of PCL-OH

25 mL of 6% solution of CTMS in toluene was added to the Schlenk flask equipped with magnetic stirring bar. The solution was removed after 24 h and the Schenk flask was dried under vacuum in 120 °C. Then, CL (6 mL, 54.14 mmol), MTEG (342 µL, 2.16 mmol) and toluene (1.80 mL, 30% vol.% of CL) were placed in reactor, degassed by two freeze-pump-thaw cycles and 6% solution of Sn(Oct)$_2$ in toluene (1.17 mL, 0.22 mmol) was added. The reaction flask was immersed in an oil bath at 100 °C. The polymerization was stopped by exposure to air after 24 h. Reaction mixture was dissolved in chloroform. The product was precipitated in methanol and dried at room temperature under vacuum to constant mass. ^{1}H NMR (600 MHz, CDCl$_3$, ppm): 4.23 (2H, -CH$_2$-O(=O)C-), 4.06 (n*2H, -CH$_2$-O(=O)C-), 3.66 (10H, 5* -O-CH$_2$-), 3.55 (2H, -CH$_2$OH), 3.38 (3H, -OCH$_3$), 2.29 (2H, -CH$_2$-COO-), 1.65 (n*4H, -CH$_2$), 1.38 (n*2H, -CH$_2$-). ^{13}C NMR (300 MHz, CDCl$_3$, ppm): 173 (C7, -COO-), 71-68 (C$_{2-5}$, -OCH$_2$-), 63 (C12, -CH$_2$-O(O=)C-), 62 (C15, -CH$_2$OH), 61 (C6, -CH$_2$-OC(=O)-), 58 (C1, -OCH$_3$), 33 (C8, -CH$_2$-C(=O)O-), 31 (C14, -CH$_2$-), 27 (C11, -CH$_2$-), 25 (C10, -CH$_2$-), 24 (C13, -CH$_2$-), 23 (C9, -CH$_2$-).

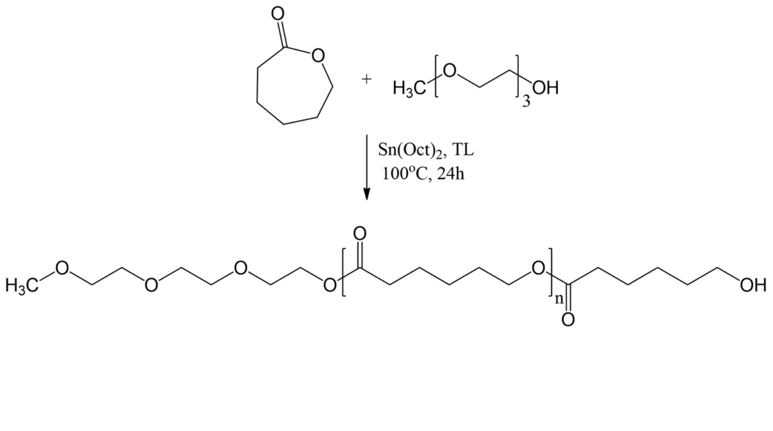

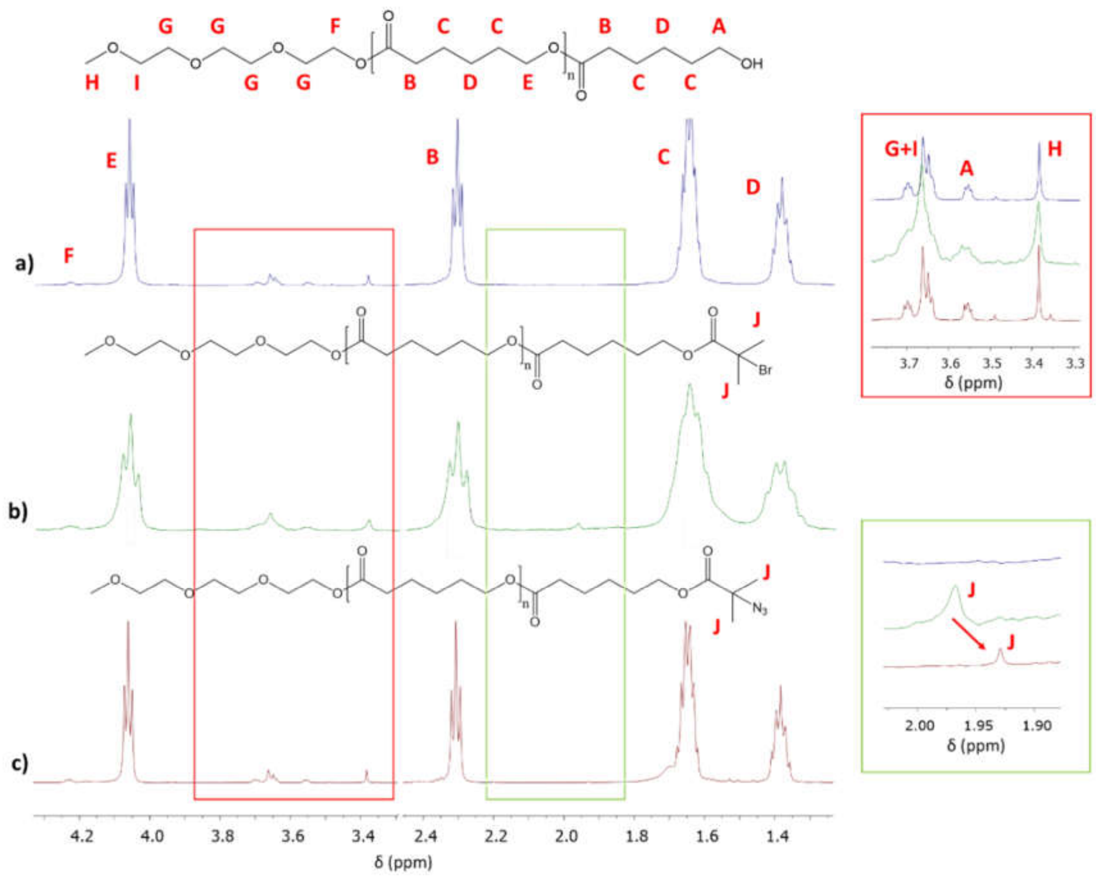

Figure S1. ^{1}H NMR spectra of (a) PCL-OH, (b) PCL-Br, and (c) PCL-N_3.

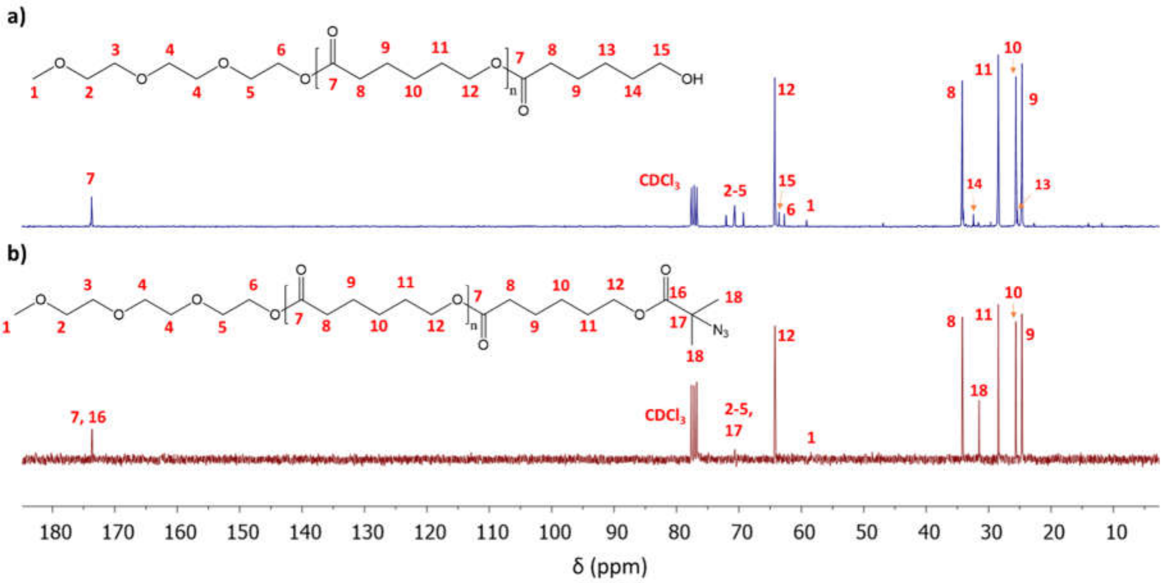

Figure S2. ^{13}C NMR spectra of (a) PCL-OH, (b) PCL-N_3.

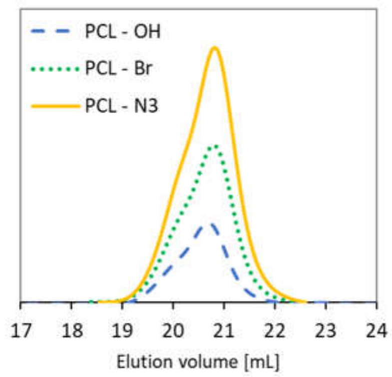

Figure S3. GPC traces for PCL4000 before and after modifications.

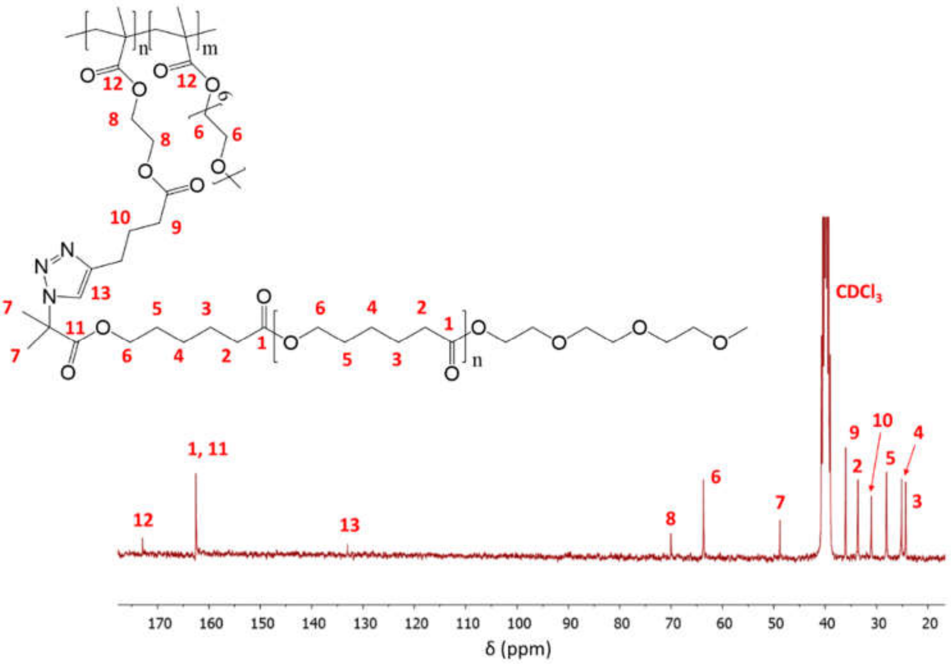

Figure S4. ^{13}C NMR of IVc_PCL4000.

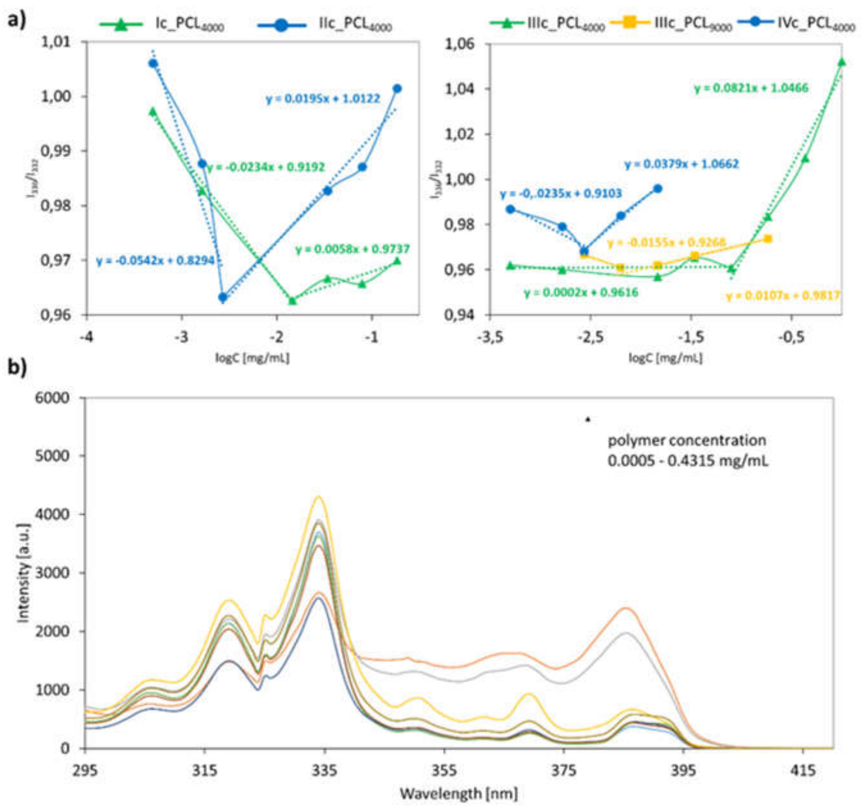

Figure S5. Representative plots of intensity I_{336}/I_{332} ratio as a function of the logarithm of copolymers concentration in aqueous solution (a) and excitation spectra of pyrene in aqueous solutions (λ = 390 nm) in dependence of IIIc_PCL$_{4000}$ copolymer concentration (b).

Table 1. Hydrodynamic diameters (D_h) for obtained micelles.

	empty		4nBRE		ARB		VitC	
	^aD_h ± SD / ^bD_h ± SD (nm)	PDI	^aD_h ± SD / ^bD_h ± SD (nm)	PDI	^aD_h ± SD / ^bD_h ± SD (nm)	PDI	^aD_h ± SD / ^bD_h ± SD (nm)	PDI
Ic_PCL$_{4000}$	272 ± 34 / 267 ± 19	1.000	279 ± 35 / 276 ± 20	0.749	453 ± 66 / 445 ± 45	0.857	588 ± 118 / 572 ± 101	0.184
IIc_PCL$_{400}$	383 ± 46 / 379 ± 24	1.000	387 ± 79 / 375 ± 65	0.662	221 ± 23 / 220 ± 20	1.000	251 ± 30 / 250 ± 14	1.000
IIIc_PCL$_{4000}$	410 ± 56 / 405 ± 35	1.000	187 ± 26 / 188 ± 17	0.824	218 ± 24 / 217 ± 8	1.000	359 ± 69 / 350 ± 57	0.710
IIIc_PCL$_{9000}$	296 ± 31 / 295 ± 30	1.000	178 ± 29 / 181 ± 22	0.641	282 ± 35 / 280 ± 19	1.000	2.5 ± 0.31 / 564 ± 84	1.000
IVc_PCL$_{4000}$	344 ± 51 / 339 ± 36	0.734	186 ± 26 / 187 ± 17	0.791	504 ± 63 / 498 ± 36	1.000	68 ± 7 / 68 ± 5	1.000
Vc_PCL$_{4000}$	256 ± 26 / 255 ± 25	1.000	238 ± 41 / 236 ± 31	0.684	253 ± 33 / 252 ± 20	1.000	566 ± 72 / 561 ± 40	0.749
Vc_PCL$_{9000}$	57 ± 6 / 57 ± 2	1.000	242 ± 53 / 241 ± 45	0.484	339 ± 52 / 334 ± 38	0.607	443 ± 60 / 437 ± 37	1.000
VIc_PCL$_{4000}$	317 ± 40 / 315 ± 23	1.000	317 ± 114 / 305 ± 99	0.508	222 ± 31 / 222 ± 21	0.967	44 ± 5 / 45 ± 2	0.663
VIc_PCL$_{9000}$	[c]13 ± 2 / [c]326 ± 27	0.756	230 ± 42 / 229 ± 33	0.224	471 ± 99 / 452 ± 84	0.615	747 ± 103 / 740 ± 68	0.380

[a] Hydrodynamic diameters (D_h) by volume; [b] hydrodynamic diameters (D_h) by intensity; [c] value of particle size for dominated fraction.

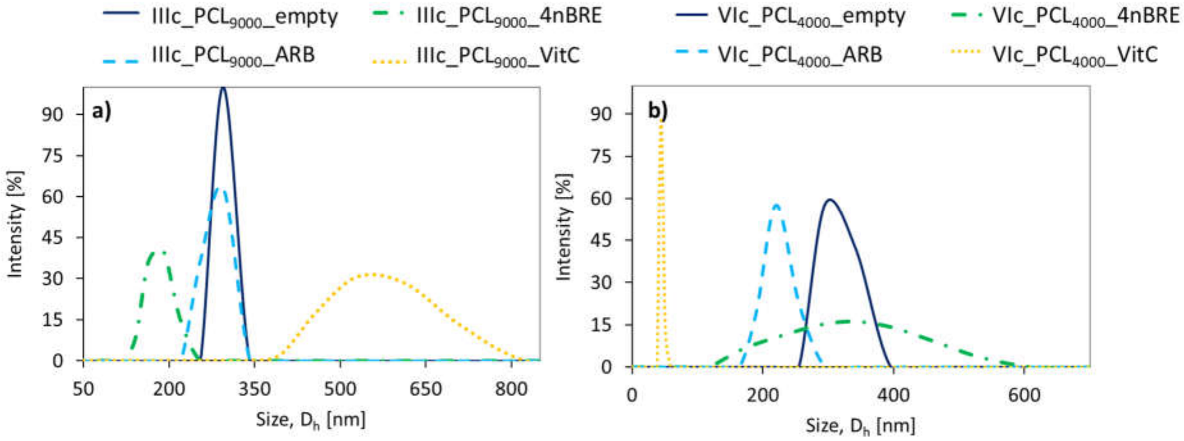

Figure S6. Size distribution intensity plots for micelles formed by (a) IIIc_PCL9000, and (b) VIc_PCL4000.

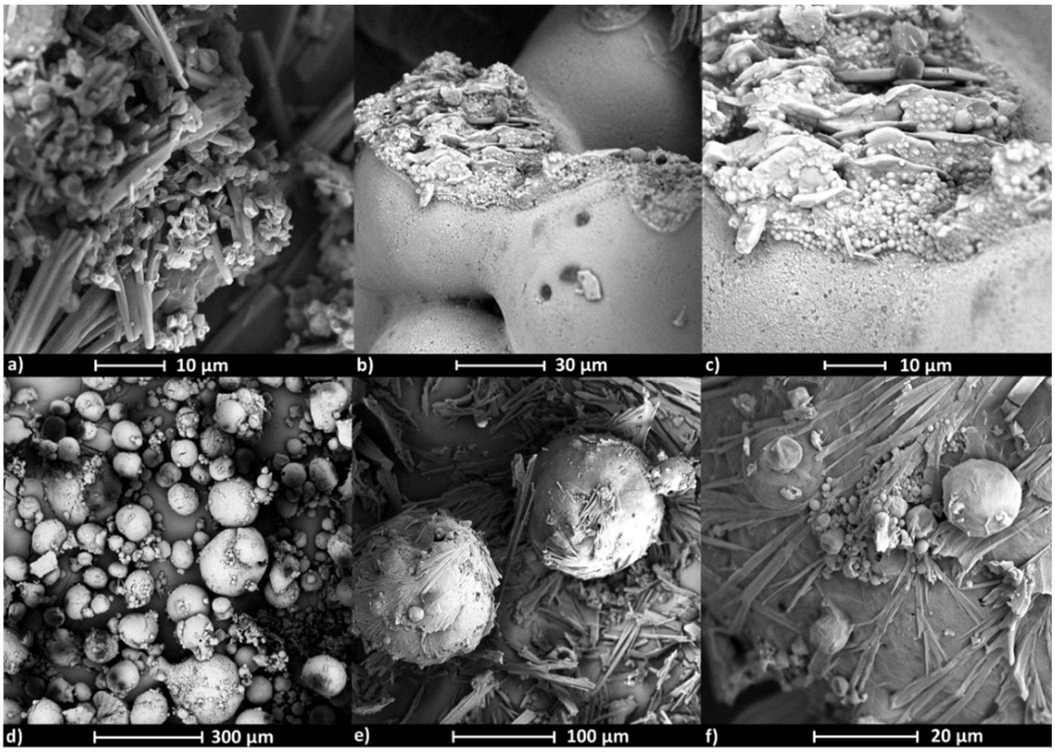

Figure S7. SEM images for micelles formed by (a) IIIc_PCL9000 copolymer with arbutin, (b, c) IIIc_PCL9000 copolymer with vitamin C, (d) VIc_PCL4000 copolymer (empty micelles) and (e, f) VIc_PCL4000 copolymer with arbutin.

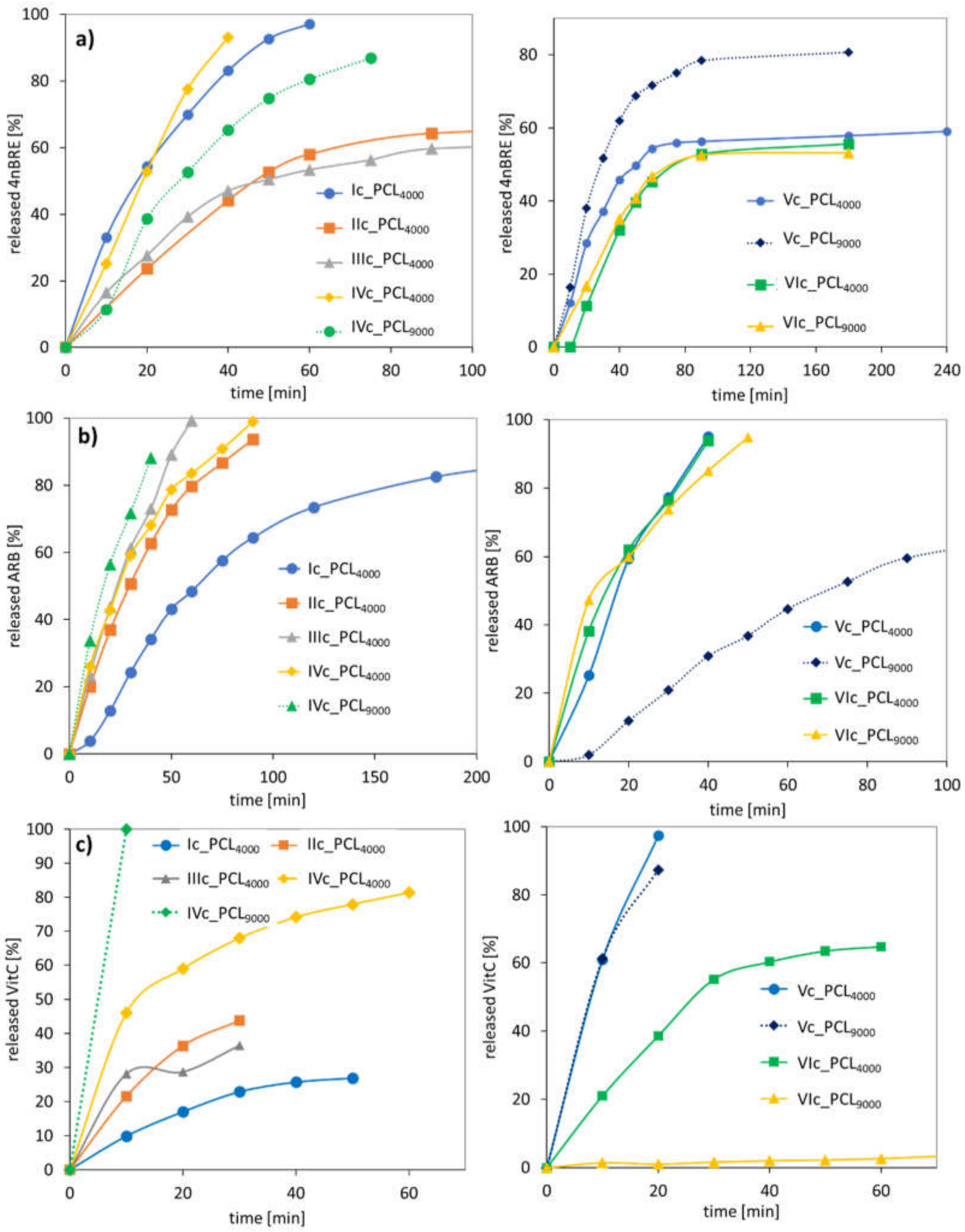

Figure S8. Kinetic profiles for (a) 4nBRE, (b) ARB, and (c) VitC released from polymer micelles at pH = 5.5.

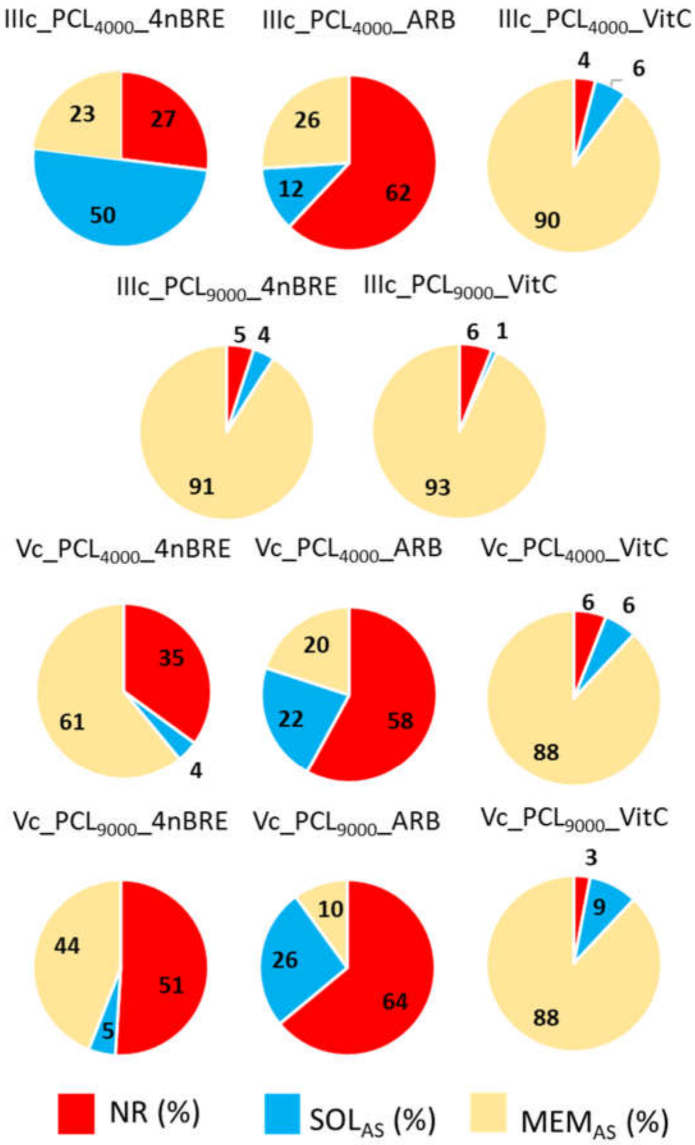

Figure S9. Graph of the amount of unreleased substance (NR), amount of released substance which passed through the membrane into the solution (SOL_{AS}), and amount of released substance that remained in the membrane (MEM_{AS}).
